# Delay in Vaccine Access in ASEAN Countries

**DOI:** 10.3390/ijerph19073786

**Published:** 2022-03-22

**Authors:** Nilubon Subsittipong, Junjeong Choi, Tae Hyun Kim, Euna Han

**Affiliations:** 1 Department of Pharmaceutical Medicine and Regulatory Sciences, College of Medicine and Pharmacy, Yonsei Institute of Pharmaceutical Sciences, Yonsei University, Seoul 03722, Korea; nilubon@yonsei.ac.kr (N.S.); junjeong@yonsei.ac.kr (J.C.); 2 Graduate School of Public Health, Yonsei University, Seoul 03722, Korea; thkim@yuhs.ac

**Keywords:** vaccine, approval lag, Asia-Pacific

## Abstract

Background: The introduction of new vaccines has been delayed in some countries in the Asia-Pacific region, which has led to delays in accessing vaccines for target patients. However, the approval lag of vaccines in the Asia-Pacific region has not been assessed. The objective of this study is to assess the availability and approval lag of vaccines in Asia-Pacific countries and compare them among Asia-Pacific countries, the United States (US), and Europe (EU). Methods: The information on vaccines prequalified by the World Health Organization (WHO) between 2010 and 2019 was obtained primarily from the WHO website. The date of approval of the WHO prequalified vaccine in Australia, India, South Korea, Thailand, Singapore, Malaysia, the US, and EU was retrieved from the official website of national regulatory agencies. The vaccines were divided into two groups based on their first approval pathway, that is, vaccines that were first approved by SRA (Stringent Regulatory Authority including the US, EU, and WHO) and those that were first approved by non-SRA. The absolute approval lag represented the availability of the vaccine. Relative approval lag represented the lag time between the approval date of the country of interest and the first global approval date and was measured as the median approval lag. A Mann–Whitney U test was used to examine statistical differences between relative approval lag between the SRA first and the non-SRA first groups. Results: A total of 92 vaccines were prequalified by the WHO between 2010 and 2019, but only 61 vaccines were included in the analysis. Over 50% of vaccines were first licensed by non-SRAs. Of all the WHO-prequalified vaccines, the median approval lag in the ASEAN countries in this study was longer than those in the US and EU, with a median of 30 months in Australia, 15 months in South Korea, 52 months in Thailand, and 23 months in Singapore, compared to 0 months in the US and EU. The differences in approval lags between SRA first vaccines and non-SRA first vaccines were statistically significant in South Korea and Thailand (*p* < 0.05). Conclusions: The approval lag of vaccines was observed in the Asia-Pacific region, indicating a gap between the Asia-pacific region and the US and EU in regard to access to new vaccines. Future studies need to analyze the background factors related to the gap in availability and vaccine approval lag in the Asia-Pacific region and assess the impact of vaccine approval lag in the region.

## 1. Introduction

Nearly two-thirds of the world’s population live in the Asia-Pacific region. The region has a high diversity in cultural, political, economic, and other areas that influence vaccine development, implementation, and registration [1,2]. In the Asia-Pacific region, vaccine manufacturers have transformed from downstream vaccine processing providers to manufacturers of more affordable, previously approved vaccines and innovators of novel vaccines [1]. The financial funding in biomedical research and development (R&D) related to vaccines in Asia Oceania increased from 18.2% of the global share to 23.8%, while it decreased in the United States (US) (by 9.1%), Canada (11.7%), and Europe (EU) (2.2%) between 2007 and 2012 [3,4]. The trend could be due to the pharmaceutical industry’s recognition of highly growing vaccine opportunities in Asia [1].

However, the transformation was not equal across countries, and high-income and low-middle income countries still have different vaccine development, implementation, and registration processes. The differences in vaccine development in the region include all steps of vaccine innovation, production, and consumption. The region includes China, India, Indonesia, and Vietnam, which are eligible countries or graduate countries from the Global Alliance for Vaccines and Immunization (GAVI), a global public–private health corporation to help poor countries obtain access to vaccines with full financial support [5]. However, most middle-income countries are not eligible for full financial funding from GAVI, leading to high vaccine prices and limited and unpredictable funding in their countries. The lack of financial resources may slow down access to new vaccines in middle-income countries [6,7].

There is a delay in the introduction of new vaccines in some countries in the region [7]. The delay is called “drug lag or approval lag”, which refers to a delay in availability in the country and is calculated from the difference between the approval date in a given country and the world’s first approval date [8,9]. Drug development and registration in Asia is commonly delayed compared to the US and EU, leading to a crucial unmet need due to restricted or delayed drug access for Asian populations [10]. There are numerous factors related to approval lag, including molecular type, therapeutic class, the status of orphan drug, registration and review procedure, nationality, the requirement for local data, and global clinical trials [11].

Asian-Pacific countries do not have a central regulatory approval process like the European Medicines Agency (EMA) in Europe or the Pan American Health Organization (PAHO), which coordinates regional vaccination programs and centrally purchases eligible vaccines [1]. The diversity in regulatory requirements and procedures delays a vaccine’s marketing authorization and consequently leads to a delay in access to safe and effective vaccines that may prevent significant morbidity and mortality in their populations [12].

There are several efforts to streamline regulatory requirements between countries and regions. There are also efforts to encourage mutual recognition practices between national regulatory authorities in an effort to preserve resources and time and avoid redundancy in Asia [12]. For example, the Association of Southeast Asian Nations (ASEAN) (ASEAN consists of Brunei-Darussalam, Cambodia, Indonesia, Lao PDR, Malaysia, Myanmar, Philippines, Singapore, Thailand, and Vietnam) introduced sub-regional regulatory harmonization to improve technical requirements and regulatory procedures. It aimed to ease marketing and technical differences across the member countries [1]. However, local regulatory agencies adjust the Common Technical Dossier (CTD) template, defeating the original objective of harmonization. Consequently, the differences in requirements are still high, particularly in structure numbering and contents, and in the registration processes [12]. This would restrict vaccine access for people in the region due to the increase in preparatory dossiers related to the same product to fulfill specific requirements for certain countries and the different timelines of each national regulatory agency for the evaluation of the submitted information [2].

Vaccines are a highly cost-effective intervention to prevent infectious diseases with rare serious adverse reactions [13]. Effective vaccination has made a great contribution to children, families, communities, economies, and global health. Vaccination not only protects people from vaccine-preventable diseases, which are the causes of millions of deaths per year, but also saves time and money for their families and reduces the social and economic burden of the disease on communities [6].

Despite the fact that understanding drug lag for vaccines is crucial for public health, there is no comparative study on the approval lag of vaccines in the Asia-Pacific region. We fill the gap in the literature and evaluate the availability and approval lag of vaccines in Asia-Pacific countries and compare among Asia-Pacific countries, the US, and EU.

## 2. Methods

### 2.1. Data

We collected information on vaccines that were prequalified by WHO between 1 January 2010, and 31 December 2019. The WHO prequalification is a systematic process to assess and ensure that healthcare products comply with international standards of quality, safety, and efficacy [14]. WHO prequalification indicates that the vaccines are appropriate for the target population and the specifications for packaging and presentation of UN countries interested in procuring that vaccine for their national immunization program [15,16,17]. Currently, low-to-middle income countries account for over 40% of all prequalified medicines manufacturers and 50% of prequalified vaccine manufacturers [18]. The coverage of vaccine prequalification is for routine immunization for the prevention of 24 priority diseases [18].

Information about commercial names, vaccine types, and dates of prequalification was retrieved from the WHO website. Information about the date of approval of the WHO prequalified vaccine in selected Asia-Pacific countries, the US FDA, and EMA was retrieved from the official websites of the drug approval agencies for each country (Table 1).

**Figure 1:**
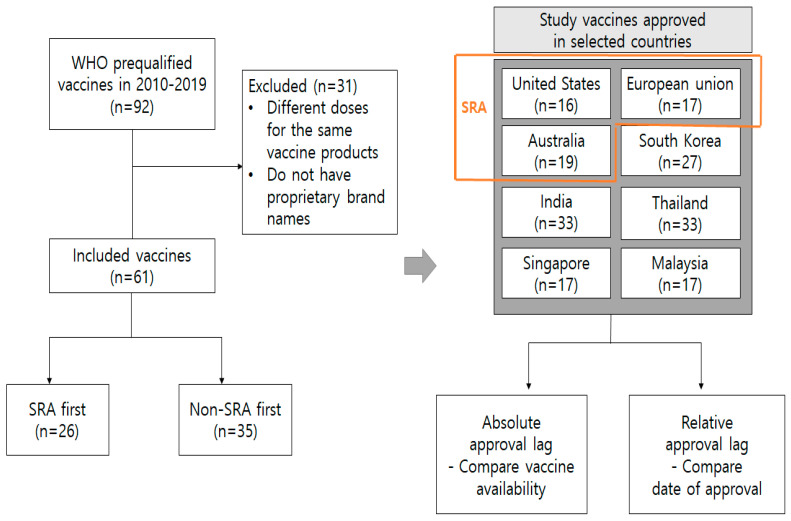
Flow diagram of method.

There were prequalified 92 prequalified vaccines between 1 January 2010 and 31 December 2019. We excluded different doses of the same vaccine products and used the fastest prequalified date among different doses of the same vaccine. We excluded vaccines that do not have proprietary brand names because of the difficulty encountered in collecting approval data and approved dates. Ultimately, 61 vaccines were evaluated in the present study (Figure 1).

### 2.2. Variables

The 61 vaccines we extracted were divided into two groups based on their first approval pathway, that is, vaccines that were first approved by the stringent regulatory authority (SRA first) (the stringent regulatory authority (SRA) Australia, Austria, Belgium, Bulgaria, Canada, Cyprus, Czech Republic, Denmark, Estonia, Finland, France, Germany, Greece, Hungary, Iceland, Ireland, Italy, Japan, Latvia, Liechtenstein, Lithuania, Luxembourg, Malta, Netherlands, Norway, Poland, Portugal, Romania, Slovakia, Slovenia, Spain, Sweden, Switzerland, United Kingdom, and United States of America [27]. Organization, W. H. List of Stringent Regulatory Authorities (SRAs). http://www.who.int/medicines/regulation/sras/en/ (accessed on 22 January 2021)) and those that were first approved by non-SRA (non-SRA first). The stringent regulatory authorities (SRA) are the national regulatory agencies, which are the members or observers, or associates of the International Council for Harmonisation of Technical Requirements for Pharmaceuticals for Human Use (ICH) [27].

The vaccine approval date refers to the date of regulatory approval by the national regulatory authorities, including the US Food and Drug Administration (US FDA), the European Medicines Agency (EMA), the Therapeutic Goods Administration in Australia, the Central Drugs Standard Control Organization (CDSCO) in India, the Ministry of Food and Drug Safety (MFDS) in South Korea, the Thai Food and Drug Administration in Thailand, the Health Sciences Authority (HSA) in Singapore, and the National Pharmaceutical Regulatory Agency (NPRA) in Malaysia.

We assessed and described the approval lag in terms of “absolute approval lag” and “relative approval lag”. Absolute approval lag is the percentage of newly approved vaccines in the countries of interest out of the total number of all studied vaccines. Relative approval lag is the delay in approval in a study country after the drug’s first approval [28].

### 2.3. Statistical Analysis

Continuous variables were shown as the median and range, and categorical variables were shown as the number and percentage. The median was used to compute drug lag, as it is less sensitive to extreme values than the average. The Mann–Whitney U test was used to examine the statistical difference between the relative approval lag between the SRA first and non-SRA first groups. A *p*-value of ≤0.05 was considered to indicate statistical significance. Statistical analysis was performed using SPSS version 25 (SPSS, Chicago, IL, USA).

## 3. Results

Characteristics of WHO-prequalified vaccines are summarized in Table 2. Of the 61 WHO-prequalified vaccines in the analysis, 15 vaccines (24.6%) are influenza vaccines and 9 (14.8%) are diphtheria–tetanus–pertussis. More than half of the vaccines were first licensed by the non-SRAs, as 21 (34.4%) were approved by the CDSCO of India and 10 vaccines (16.4%) were approved by the MFDS of South Korea. Approximately 42.6% of the studied vaccines were approved in the SRA first, including 14 vaccines (23%) by the US FDA and 12 vaccines (19.7%) by EMA and EU countries (Table 2).

The absolute approval lags of the studied vaccines are shown in Table 3. Half of the studied vaccines were approved in Thailand (33 vaccines, 54%) and India (33 vaccines, 54%), followed by South Korea (27 vaccines, 44%). The majority of Asian countries approved more studied vaccines, i.e., WHO prequalified, than the US and the EU. The US approved the studied WHO prequalified vaccines only from the SRA first, whereas other countries approved vaccines from both SRA first and non-SRA first.

Vaccines first approved and manufactured in the US or the EU are available in all selected countries. Those approved and manufactured in India are available in all selected Asian countries and EU, and those approved and manufactured in South Korea are licensed only in their own country and ASEAN, including Thailand, Singapore, and Malaysia (Table 3).

Diphtheria–tetanus–pertussis, hepatitis A, influenza A meningococcal, pneumococcal, and rotavirus vaccines were licensed in all selected countries. Among all vaccine types, influenza vaccines accounted for the largest proportion of the studied WHO-prequalified vaccines (15 vaccines, 24.6%). South Korea approved 60% of the studied WHO-prequalified influenza vaccines (9 vaccines, 15%). The Ebola vaccine was only approved in the US and EU, while Japanese encephalitis vaccines were only approved in Asian countries (Table 3).

**Figure 2:**
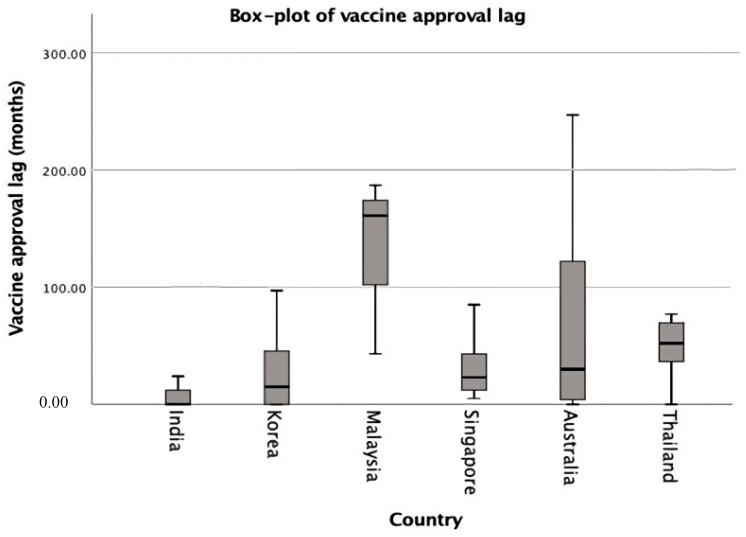
Vaccine relative approval lag.

**Figure 3:**
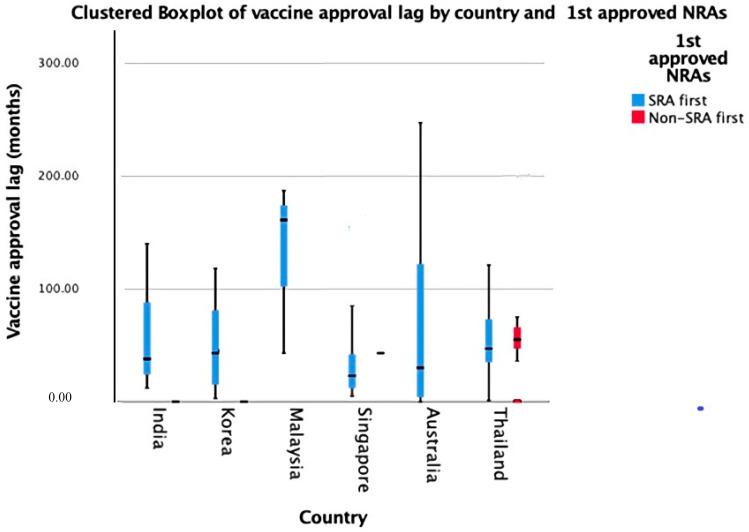
Boxplot of vaccine relative approval lag divided by the first approval national regulatory agencies.

Relative approval lags of the studied WHO prequalified vaccines are summarized in Figure 2 and Figure 3. Among all countries, Malaysia has the longest relative approval lag with 161 months (13.4 years) being lagged from the world’s first approval date (Figure 2). Among the non-SRA first vaccines, the longest approval lag was 55 months (4.5 years) in Thailand. The approval lag in the non-SRA first group in South Korea was shorter than that in the SRA group. This may be because all of the non-SRA first vaccines in South Korea were first approved in South Korea, so there was no approval lag for the non-SRA first vaccines. However, there was an approval lag in the SRA’s first vaccines because they were approved from outside South Korea. This requires time to prepare Korea-specific dossiers for submission and collect local data in South Korea before approval. On the other hand, the approval lag in the SRA first group in Thailand is shorter than that in the non-SRA first group (Figure 3).

**Figure 4:**
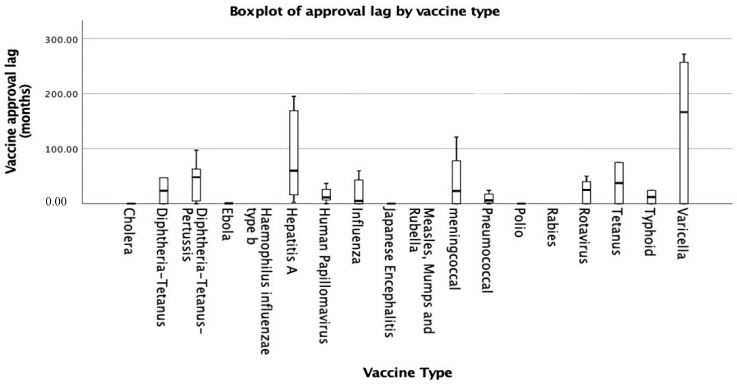
Clustered boxplot of vaccine relative approval lag divided by the first approval NRAs.

The relative approval lag of WHO prequalified vaccines by vaccine type is shown in Figure 4. Among all vaccine types, varicella vaccines have the longest median approval lag with 272 months, whereas pneumococcal vaccines have the shortest median approval lag with 8.5 months The EU and US mostly approve their own vaccines as India does, and thus, we have no elements to know how long would take the evaluation of non-SRA first vaccines in EU and US (Figure 4).

The difference in relative approval lag between the SRA first and non-SRA first groups was examined only in India, South Korea, and Thailand because non-SRA first vaccines were not available in the US and because of the lack of approval date data for non-SRA first vaccines in other countries. Our findings showed that the difference between the relative approval lag between SRA first and non-SRA first groups in South Korea and Thailand is statistically significant (Table 4).

## 4. Discussion

This study aims to evaluate the availability of vaccines and the delay in access to vaccines in Asia-Pacific countries. Our results found that the non-SRA first vaccines were over a half of the WHO-prequalified vaccines, with 34.4% and 16.4% being first approved by India and South Korea, respectively. Usually, the vaccine manufacturing country is the first country to approve the vaccines. India and South Korea are members of the Developing Countries Vaccine Manufacturers Network (DCVMN), which has the capacity to manufacture and supply high-quality vaccines at affordable prices. The DCVMN plays an important role in supplying vaccines in developing countries [29], which are the target countries for WHO-prequalified vaccines. However, the vaccines from India and South Korea, which are non-SRA [27], may have the difficulty in the approval process from SRA due to the strict requirements and registration process.

For the relative approval lag of all vaccines and SRA-first vaccines, the Asia-Pacific countries have a longer relative approval lag than the US and the EU. Our finding of the longer approval lag in Asia compared to the US and EU was consistent with previous studies that assessed the approval lag in cancer drugs in India [30], inflammatory bowel disease drugs in selected Asian countries (Japan, China, South Korea, Taiwan, and the Philippines) [28], and cardiovascular drugs in India [31]. The approval lag of vaccines in Asia may be because of the differences in vaccine regulatory requirements and processes in Asia-Pacific countries, compared to the US and EU. South Korea requires local clinical study data for review of safety and efficacy of biological products, including vaccines [32]. Local clinical data help to identify racial differences and establish the optimal dosage [33], but it takes time to conduct local clinical trials and delays the approval of vaccines in Korea. However, global clinical trials can eliminate the need to conduct the new local clinical trials for approval [28]. In the past decades, the number of global clinical trials conducted in Korea has grown rapidly, which might help reduce drug approval lag [34].

Southeast Asian countries, such as Thailand, Malaysia, and Singapore, use the ASEAN (the Association of Southeast Asian Nations) Common Technical Dossier (CTD) format [24,25,26], which aims to streamline regulatory requirements between countries and regions to preserve resources and time and avoid redundancy in ASEAN [1]. Dellepiane et al. [12] compared the CTD numbering structure and contents for vaccine registration from Asian countries to the ICH CTD, which is commonly used in the ICH member countries, including the US, EU, Australia, and South Korea. The comparison between the ASEAN and the ICH CTD showed a 93% similarity in content, but 100% differences in numbering because the dossier structures are different. The comparison with the ICH CTD showed only a 25% similarity for content and a 75% difference for the Thailand CTD and a 24% similarity in content and an 83% difference for the India CTD. The difference in these CTDs from the ICH CTD may be one of contributing factors for the vaccine approval lag. Especially, Thailand CTD is different in contents and numbering from both the ASEAN CTD and the ICH CTD. The difference in content requirements influences the registration timeline, and thus, pharmaceutical companies need more time to prepare a country-specific CTD for vaccine registration, leading to unnecessary efforts, prolonged regulatory processes, and delayed access to vaccines in the country [12].

The approval lag in the SRA first group in Thailand is shorter than those in the non-SRA first group. This may be because the SRA is more strict in requirements and review for approval than non-SRA, and, in Thailand, pharmaceutical products that have already been approved by the SRA can use an accelerated process to speed up the registration of vaccines [35]. This was similarly reported in Ahonkhai et al. [36]; they divided vaccines by their first approval paths, SRA first versus non-SRA first, in Sub-Sahara Africa, and reported that the approval time of SRA first vaccines was shorter than those approved by non-SRA first (median 11 vs. 18 months).

Pneumococcal vaccines have the shortest median approval lag. Pneumococcal infection is one of the major vaccine-preventable diseases [37] and a major public health issue worldwide, accounting for 15% of all deaths of children younger than 5 years old [38]. The burden of pneumococcal infection is high in Asia, with high mortality and high incidence, particularly in South Asia [39], which might explain our findings of the shortest approval lag for pneumococcal vaccine. Among all vaccine types, varicella vaccines have the longest median approval lag. Varicella is considered a mild disease, especially in healthy children, but can cause severe and fatal complications [40]. However, the awareness of varicella is low, and also, it has not received significant global or regional attention, particularly in low- to middle-income countries [41]. The varicella vaccine is not one of the target vaccines in Gavi’s current vaccine investment strategy despite the high disease burden of varicella in the region in the Asia-Pacific region [7].

Due to the difficulty of collecting vaccine approval data and approval dates, some data may be missing, especially for Malaysia and India. These countries have no online searchable databases for vaccines; the approval data from India was obtained from a summary of product characteristics, and the approval data from Malaysia was obtained from a Registered Product List. The challenge in data collection resulted in the small sample size in the analysis. Therefore, the relative drug lag in some countries might not represent the actual relative drug lag in those countries. The analysis of possible reasons behind the delays in approval of vaccines in the Asia-Pacific region is not conducted in this study, due to the limitation of data available. The same question needs to be addressed for vaccine approval for coronavirus disease (COVID-19) in future studies.

The SRA has more facilities and experts to review vaccines for marketing authorization compared to the non-SRA. The marketing authorization in SRA is usually faster than in non-SRA, and thus, people in the SRA can get access to vaccines faster than in the non-SRA. At the same time, the case of Australia and India need further attention. India has quick access to the vaccines it manufactures while Australia has a slow marketing authorization process compared to India, Singapore, or Korea (Figure 2 and Figure 3). This observation is true for SRA-first vaccines since Australia mostly approves SRA-first vaccines. Moreover, non-SRA countries generally approve more vaccines than the US and EU (about 57% of the whole available vaccines). Australia also shows a wider range of approval lags for SRA first vaccines compared to other Asia-Pacific countries investigated. The observed disparities might stem from either manufacturers and/or national public health priorities. Future research would need to specify at what moments the manufacturers deposited their dossiers to the national regulatory agencies for approval to identify sources of the approval lag and improve the lag accordingly.

Regardless, our study has merit by showing the approval lag of vaccines in the Asia-Pacific region, indicating a gap between the Asia-Pacific region and the US and EU in regard to access to new vaccines. Vaccination is a cost-effective intervention and one of the most successful methods to prevent infectious diseases and improve health status. Access to vaccines can also save time and money and reduce the social and economic burden of the disease. Therefore, continued efforts to reduce the approval lag for vaccines are essential for public health in the region. Future studies need to analyze the background factors related to the gap in availability and vaccine approval lag in the Asia-Pacific region and assess the impact of vaccine approval lag in the region.

## 5. Conclusions

The gap in vaccine availability may be attributed to the incidence and disease burden, financial resources, and inclusion in national immunization programs. The vaccine approval lag in the Asia-Pacific region may be attributed to the differences in vaccine development and registration process in between countries and regions, low disease attention and awareness, low disease incidence and burden in the region, and the non-national immunization programs vaccines.

## Figures and Tables

**Table 1:** Data sources.

Region	Country	Regulatory Agency	Data sources
North America Europe	United States	US Food and Drug Administration (FDA)	The Center for Biologics Evaluation and Research (CBER), Vaccines Licensed for Use in the United States: https://www.fda.gov/vaccines-blood-biologics/vaccines/vaccines-licensed-use-united-states (accessed on 10 June 2021) [19]
	European Union	European Medicines Agency (EMA)	Committee for Medicinal Products for Human Use (CHMP), The European Public Assessment Report (EPAR): https://www.ema.europa.eu/en (accessed on 10 June 2021) [20]
Australia	Australia	Therapeutic Goods Administration (TGA)	the Australian Register of Therapeutic Goods (ARTG).; Australia: https://tga-search.clients.funnelback.com/s/search.html?query=&collection=tga-artg (accessed on 10 June 2021) [21]
South Asia	India	The Central Drugs Standard Control Organization (CDSCO)	Vaccines, SmPC; https://cdsco.gov.in/opencms/opencms/en/biologicals/Vaccines/ (accessed on 10 June 2021) [22]
East Asia	South Korea	Ministry of Food and Drug Safety	Drug search; https://nedrug.mfds.go.kr/searchDrug (accessed on 10 June 2021) [23]
South East Asia	Thailand	Thai Food and Drug Administration	Product search; https://porta.fda.moph.go.th/FDA_SEARCH_ALL/MAIN/SEARCH_CENTER_MAIN.aspx (accessed on 10 June 2021) [24]
	Singapore	Health Sciences Authority (HSA)	Listing of Registered Therapeutic: https://data.gov.sg/dataset/listing-of-registered-therapeutic-products (accessed on 10 June 2021) [25]
	Malaysia	National Pharmaceutical Regulatory Agency (NPRA), Ministry of Health Malaysia	Malaysia Drug Control Authority, Product search: https://www.npra.gov.my/index.php/en/ (accessed on 10 June 2021) [26]

**Table 2:** Characteristics of WHO-prequalified vaccines between 2010 and 2019 (*n* = 61).

Variables	Total	
	*n*	%
Total	61	100
First approval country		
Stringent Regulatory Authority first	26	42.6
United States	14	23.0
European Union	12	19.7
Non-Stringent Regulatory Authority first	35	57.4
India	21	34.4
Korea	10	16.4
China	1	1.6
Thailand	1	1.6
Indonesia	1	1.6
Cuba	1	1.6
Vaccine type		
Cholera	3	4.9
Diphtheria–tetanus	1	1.6
Diphtheria–tetanus–pertussis	9	14.8
Ebola	1	1.6
Hemophilus influenzae type b	1	1.6
Hepatitis A	3	4.9
Human papillomavirus	1	1.6
Influenza	15	24.6
Japanese encephalitis	2	3.3
Measles, mumps, and rubella	1	1.6
Meningococcal	5	8.2
Pneumococcal	3	4.9
Polio	6	9.8
Rabies	3	4.9
Rotavirus	3	4.9
Tetanus	1	1.6
Typhoid	2	3.3
Varicella	2	3.3
Year		
2010	9	14.8
2011	6	9.8
2012	3	4.9
2013	6	9.8
2014	7	11.5
2015	5	8.2
2016	4	6.6
2017	4	6.6
2018	9	14.8
2019	8	13.1

**Table 3:** Absolute approval lag of WHO-prequalified vaccine by first approval country and vaccine type (*n* = 61).

Variables	Total	US	EU	Australia	India	South Korea	Thailand	Singapore	Malaysia
	*n* (%)	*n* (%)	*n* (%)	*n* (%)	*n* (%)	*n* (%)	*n* (%)	*n* (%)	*n* (%)
Total	61 (100)	16 (26)	17 (28)	19 (31)	33 (54)	27 (44)	33 (54)	17 (28)	17 (28)
First approval		14 (23)	12 (20)		21 (34)	10 (1.6)	1 (1.6)		
First approval national regulatory agencies									
SRA first	26 (42.6)								
US	14 (23.0)	14 (20)	2 (9.8)	11 (18)	5 (8.2)	9 (15)	10 (16)	10 (16)	8 (13)
EU	12 (19.7)	2 (6.6)	12 (13)	7 (11)	7 (11)	7 (11)	7 (11)	5 (8.2)	6 (9.8)
Non-SRA first	35 (57.4)								
India	21 (34.4)		3 (4.9)	1 (1.6)	21 (34)	1 (1.6)	9 (15)	1 (1.6)	1 (1.6)
Korea	10 (16.4)					10 (1.6)	4 (6.6)	1 (1.6)	2 (3.3)
China	1 (1.6)						1 (1.6)		
Thailand	1 (1.6)						1 (1.6)		
Indonesia	1 (1.6)						1 (1.6)		
Cuba	1 (1.6)								
Vaccine type									
Cholera	3 (4.9)		1 (1.6)		1 (1.6)	2 (3.3)	1 (1.6)		1 (1.6)
Diphtheria–tetanus	1 (1.6)				1 (1.6)		1 (1.6)		
Diphtheria–tetanus–pertussis	9 (14.8)	2 (3.3)	2 (3.3)	3 (4.9)	7 (11)	4 (6.6)	8 (13)	3 (4.9)	3 (4.9)
Ebola	1 (1.6)	1 (1.6)	1 (1.6)						
Hepatitis A	3 (4.9)	2 (3.3)	2 (12)	2 (3.3)	2 (3.3)	1 (1.6)	2 (3.3)	2 (3.3)	2 (3.3)
Human papillomavirus	1 (1.6)	1 (1.6)	1 (1.6)	1 (1.6)		1 (1.6)	1 (1.6)	1 (1.6)	1 (1.6)
Influenza	15 (24.6)	4 (6.6)	1 (1.6)	3 (4.9)	3 (4.9)	9 (15)	6 (9.8)	2 (3.3)	1 (1.6)
Japanese encephalitis	2 (3.3)				1 (1.6)		1 (1.6)		
Measles, mumps, and rubella	1 (1.6)		1 (1.6)	1 (1.6)	1 (1.6)	1 (1.6)	1 (1.6)		1 (1.6)
Meningococcal	5 (8.2)	2 (3.3)	2 (3.3)	3 (4.9)	3 (4.9)	2 (3.3)	3 (4.9)	3 (4.9)	3 (4.9)
Pneumococcal	3 (4.9)	1 (1.6)	2 (3.3)	2 (3.3)	3 (4.9)	2 (3.3)	1 (1.6)	2 (3.3)	3 (4.9)
Polio	6 (9.8)		3 (4.9)		4 (6.6)	2 (3.3)	1 (1.6)		
Rabies	3 (4.9)			1 (1.6)	3 (4.9)		2 (3.3)	1 (1.6)	
Rotavirus	3 (4.9)	1 (1.6)	1 (1.6)	1 (1.6)	3 (4.9)	1 (1.6)	2 (3.3)	1 (1.6)	1 (1.6)
Tetanus	1 (1.6)				1 (1.6)		1 (1.6)		
Typhoid	2 (3.3)	1 (1.6)		1 (1.6)	1 (1.6)		1 (1.6)	1 (1.6)	
Varicella	2 (3.3)	1 (1.6)		1 (1.6)		2 (3.3)	1 (1.6)	1 (1.6)	1 (1.6)

**Table 4:** Relative approval lag for WHO prequalified vaccines divided by the first approval NRAs. (*n* = 61).

	Median Relative Approval lag ^a^, Months (Range)					
	Australia (*n* = 19)	India (*n* = 12)	South Korea (*n* = 19)	Thailand (*n* = 34)	Singapore (*n* = 17)	Malaysia (*n* = 4)
All vaccines	30 (0–247)	0 (0–140)	15 (0–257)	52 (0–272)	23 (5–240)	161 (43–187)
SRA first	30 (0–247)	38 (12–140)	43 (3–257)	47 (5–240)	23 (5–240)	161 (43–187)
Non-SRA first	na	0 (0–0)	0 (0–37)	55 (0–195)	na	na
*p*-value ^b^						
Median	na	1	0.036	0.000	na	na
Distribution	na	0.796	0.000	0.000	na	na

^a^ Relative approval lag: an index of the delay in approval in a given country after the drug’s first approval. ^b^ *p*-values were derived using the Mann–Whitney U test to examine the statistical difference between relative approval lag between SRA first and non-SRA first groups, and a *p*-value of ≤0.05 was considered to indicate statistical significance. na: not applicable due to the date limitation.

## Data Availability

Not applicable.
